# Correction: Ostermann et al. Development and Up-Scaling of Electrochemical Production and Mild Thermal Reduction of Graphene Oxide. *Materials* 2022, *15*, 4639

**DOI:** 10.3390/ma17133323

**Published:** 2024-07-05

**Authors:** Markus Ostermann, Peter Velicsanyi, Pierluigi Bilotto, Juergen Schodl, Markus Nadlinger, Guenter Fafilek, Peter A. Lieberzeit, Markus Valtiner

**Affiliations:** 1 Centre for Electrochemical Surface Technology, CEST GmbH, A-2700 Wiener Neustadt, Austria; pv@zitt.at (P.V.); juergen.schodl@cest.at (J.S.); markus.nadlinger@cest.at (M.N.); markus.valtiner@cest.at (M.V.); 2 Institute of Chemical Technologies and Analytics, Vienna University of Technology, A-1040 Vienna, Austria; guenter.fafilek@tuwien.ac.at; 3 Institute of Physical Chemistry, University of Vienna, A-1090 Vienna, Austria; peter.lieberzeit@univie.ac.at; 4 Applied Interface Physics, Vienna University of Technology, A-1040 Vienna, Austria

## Error in Figure

In the original publication [1], there was a mistake in Figure 1. In Figure 1d; the color scheme of the shown samples (an untreated graphite rod and a graphite rod pretreated anodically in 1 M NaOH for 10 min) was inversed as stated in the figure caption. The corrected Figure 1 appears below.

## Reference

The authors further wish to revise reference 1 with the corrected format: 1. Directorate-General for Research and Innovation (European Commission). *European Green Deal: Research & Innovation Call*; Publications Office: Luxembourg, 2021.

The authors apologize for the mistake and state that the scientific conclusions are unaffected. This correction was approved by the Academic Editor. The original publication has also been updated.

## Figures and Tables

**Figure 1:**
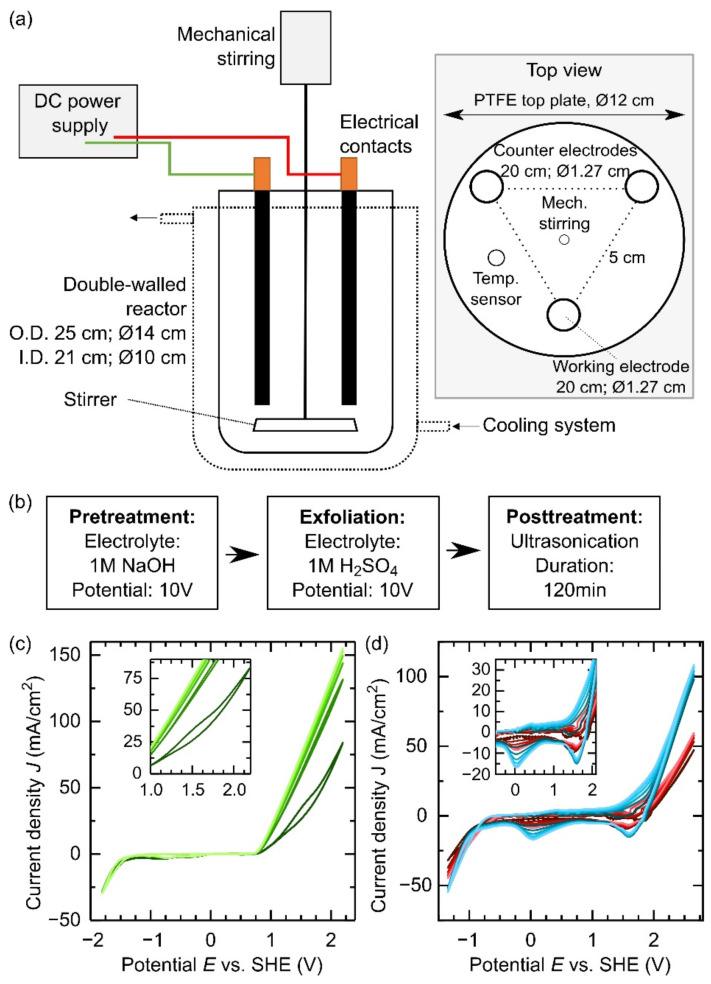
(**a**) Front (**left**) and top (**right**) sides of the electrochemical exfoliation set-up. The lateral view shows the cooling system and the power supply while the top view pictures the electrodes’ position in the electrochemical reactor. The dimensions refer to a 1600 mL reactor. (**b**) Production protocol for the up-scaling process with anodic pretreatment in 1 M NaOH. (**c**) Cyclic voltammetry applied to a graphite rod in 1 M NaOH (scan rate 10 mV/s; 6 cycles: dark green to light green). (**d**) Cyclic voltammetry applied to a graphite rod in 1 M H_2_SO_4_ (scan rate 10 mV/s; 6 cycles: dark color to light color). Red indicates an untreated graphite rod and blue indicates a graphite rod pretreated anodically in 1 M NaOH for 10 min.
