# Supplementary material for: Development and Up-Scaling of Electrochemical Production and Mild Thermal Reduction of Graphene Oxide
Source: Materials (Basel). 2022 Jul 1;15(13):4639. doi: 10.3390/ma15134639 (PMC9267235; doi:10.3390/ma15134639)
Supplement: Supplementary file 1 [file materials-15-04639-s001.zip › materials-1755990-supplementary.pdf]

Supplementary Materials: Development and up-scaling of electrochemical production and mild thermal reduction of graphene oxide

Markus Ostermann [1,†,*], Peter Velicsanyi [1,2,†], Pierluigi Bilotto [1,*], Juergen Schodl [1], Markus Nadlinger [1], Guenter Fafilek [3], Peter Lieberzeit [4] and Markus Valtiner [1,5]

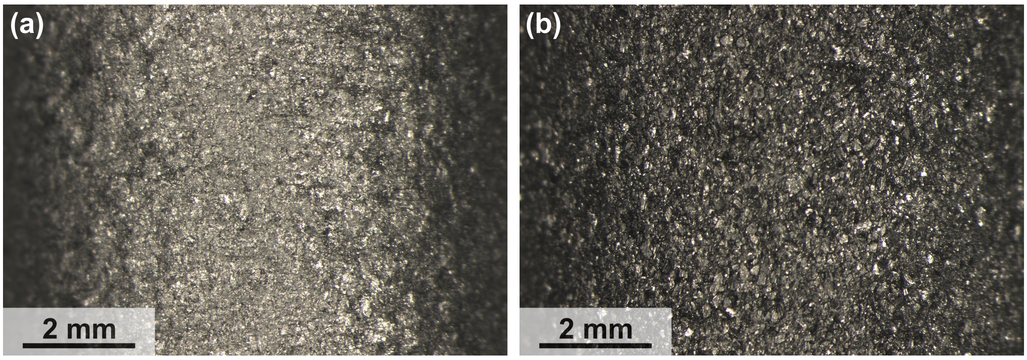

Figure S1. Optical micrographs of (a) an untreated graphite rod; (b) a graphite rod pretreated anodically in 1 M NaOH for 10 min.

Table S1. Powder conductivity σ of the graphite educt and graphene oxide powders with different pretreatment steps.

Sample	Powder conductivity σ [S/m]
Graphite educt	$3.37 \cdot 10^3$
GO - w/o pretreatment	$7.72 \cdot 10^2$
GO - NaOH anod.	$4.68 \cdot 10^2$
GO - LiOH cath.	$1.30 \cdot 10^2$
GO - NaOH cath.	$2.43 \cdot 10^2$
GO - KOH cath.	$8.54 \cdot 10^1$
GO - NH_4OH cath.	$9.55 \cdot 10^1$
GO - Li_2SO_4 add.	$7.73 \cdot 10^1$

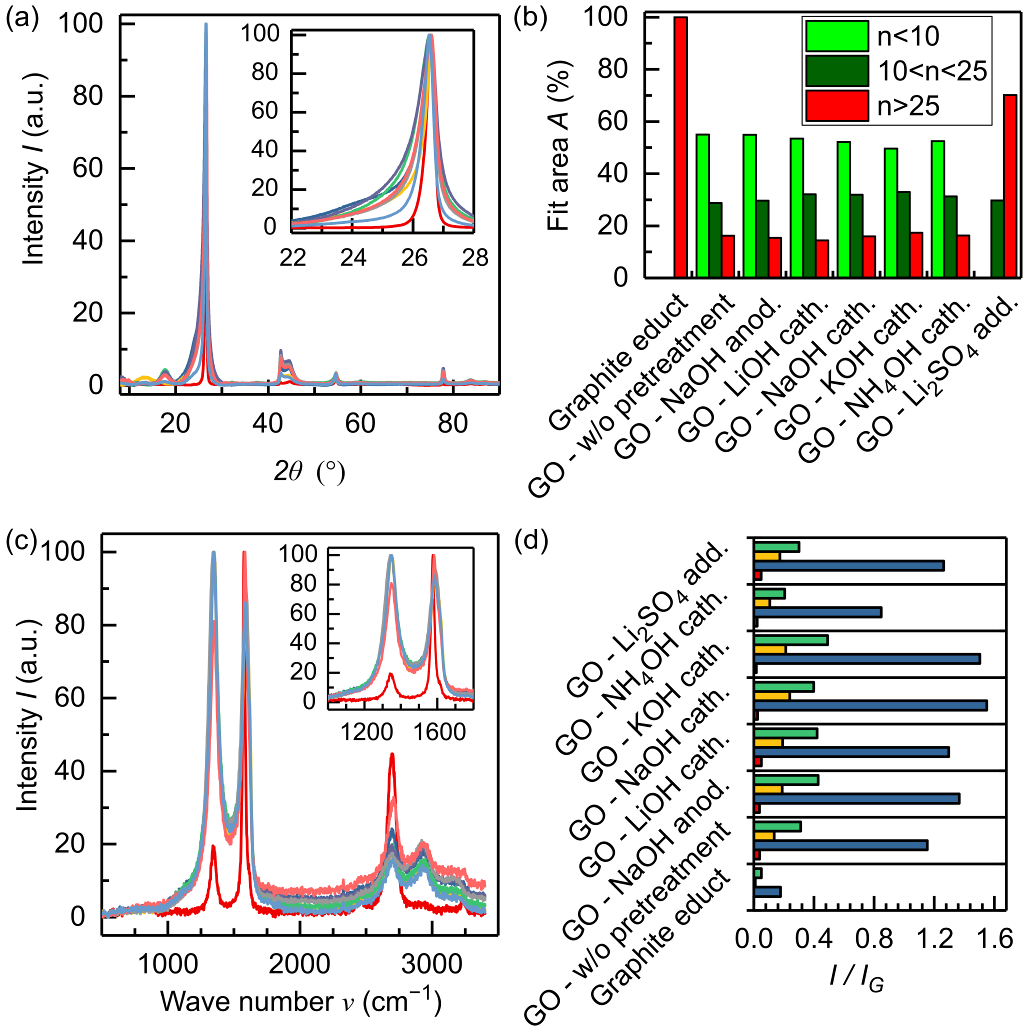

Figure S2. Comparison of different electrolytes used during pretreatment: (a) XRD diffractograms of the graphite educt (red) and different graphene oxide powders (GO without pretreatment (blue), GO with anodic NaOH pretreatment (yellow), GO with cathodic LiOH pretreatment (green), GO with cathodic NaOH pretreatment (violet), GO with cathodic KOH pretreatment (grey), GO with cathodic NH_4OH pretreatment (salmon), GO with Li_2SO_4 addition during exfoliation (light blue)); (b) calculated distribution of layers according to the fitting of the (002) reflex; (c) Raman spectra of the graphite educt (red) and different graphene oxide powders (same colour code as figure (a)); (d) calculated Intensity ratio to the G band I/I_G of 1st order defect bands (D* red, D blue, D″ yellow, D′ green).

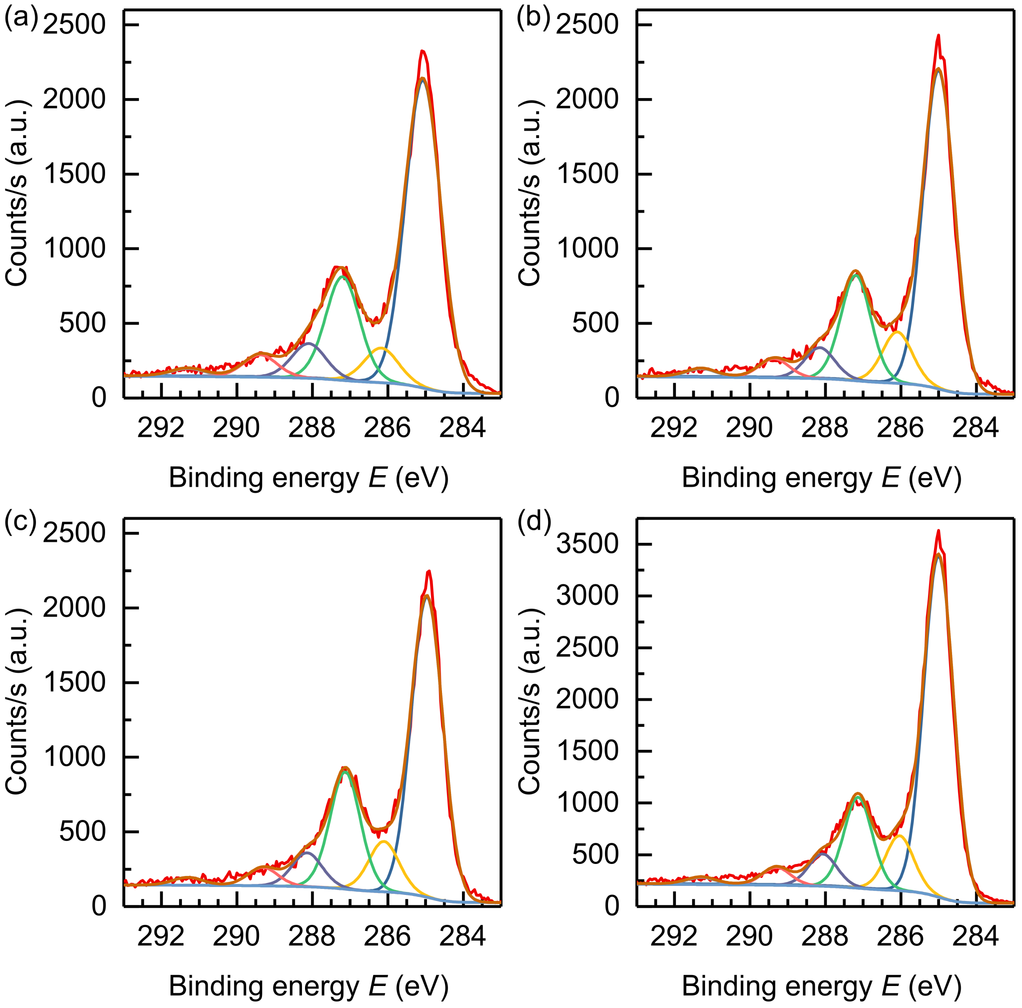

Figure S3. Fitted C1s peak in the XPS spectra (raw data (red), C-C/C-H (blue), C-OH (yellow), C-O-C (green), O-C-O/C=O (violet), O=C-O (grey), pi-pi*(salmon)) of the GO powder produced in: (a) a 500 ml reactor; (b–d) a 1600 ml reactor (Batch I (b), Batch II (c), Batch III (d))

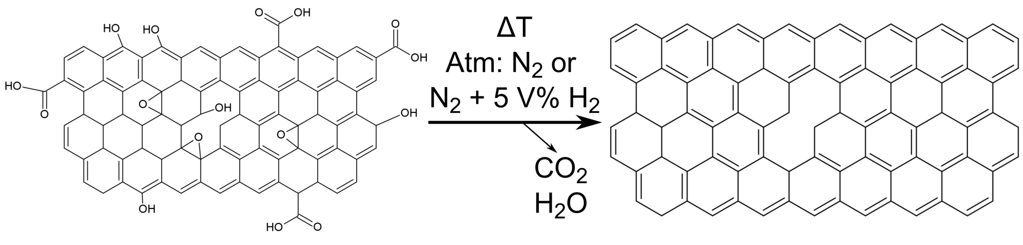

Figure S4. Scheme of thermal reduction: Thermal decomposition of oxidic functional groups in inert or reducing atmosphere under the seperation of CO_2 and H_2O.

Table S2. Set of parameters and measured powder conductivity for the individual runs of the 2-factorial screening design of the thermal reduction (Runs 1-20) and additional runs for determination of the reduction temperature influence (Runs 21-26).

Run	Temperature T_R [°C]	Time t [min]	H_2 Addition V_{H_2} [V%]	Powder Conductivity σ [S/m]
1	600	180	5	$1.89 \cdot 10^3$
2	600	180	0	$1.87 \cdot 10^3$
3	350	30	0	$1.69 \cdot 10^3$
4	475	105	0	$1.88 \cdot 10^3$
5	475	105	5	$1.81 \cdot 10^3$
6	350	30	5	$1.54 \cdot 10^3$
7	600	30	0	$1.76 \cdot 10^3$
8	600	30	5	$1.85 \cdot 10^3$
9	350	180	0	$1.59 \cdot 10^3$
10	350	180	5	$1.59 \cdot 10^3$
11	475	105	0	$1.47 \cdot 10^3$
12	600	180	0	$1.70 \cdot 10^3$
13	350	30	0	$1.52 \cdot 10^3$
14	350	180	0	$1.64 \cdot 10^3$
15	600	30	5	$1.61 \cdot 10^3$
16	475	105	5	$1.57 \cdot 10^3$
17	350	30	5	$1.41 \cdot 10^3$
18	350	180	5	$1.39 \cdot 10^3$
19	600	30	0	$1.74 \cdot 10^3$
20	600	180	5	$1.73 \cdot 10^3$
21	800	30	0	$2.31 \cdot 10^3$
22	900	30	0	$2.42 \cdot 10^3$
23	150	30	0	$7.48 \cdot 10^2$
24	250	30	0	$1.55 \cdot 10^3$
25	130	30	0	$5.29 \cdot 10^2$
26	110	30	0	$3.93 \cdot 10^3$

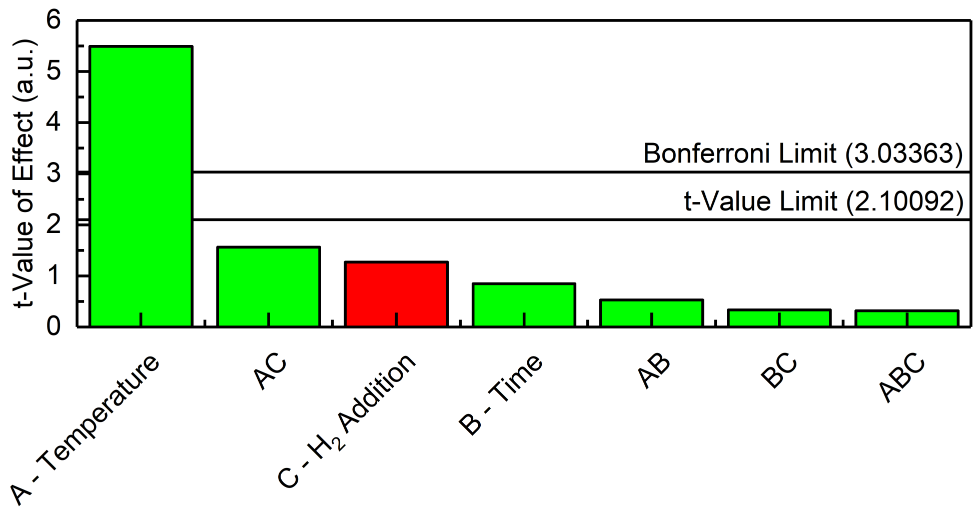

Figure S5. Pareto chart of thermal reduction screening design showing the standardized effect of each parameter (A - reduction temperature T_R, B - time t, C - H_2 addition V_{H_2}) and the combination of parameters (AB, AC, BC, ABC) on the resulting powder conductivity σ; green implies a positive effect on the powder conductivity and red a negative effect.

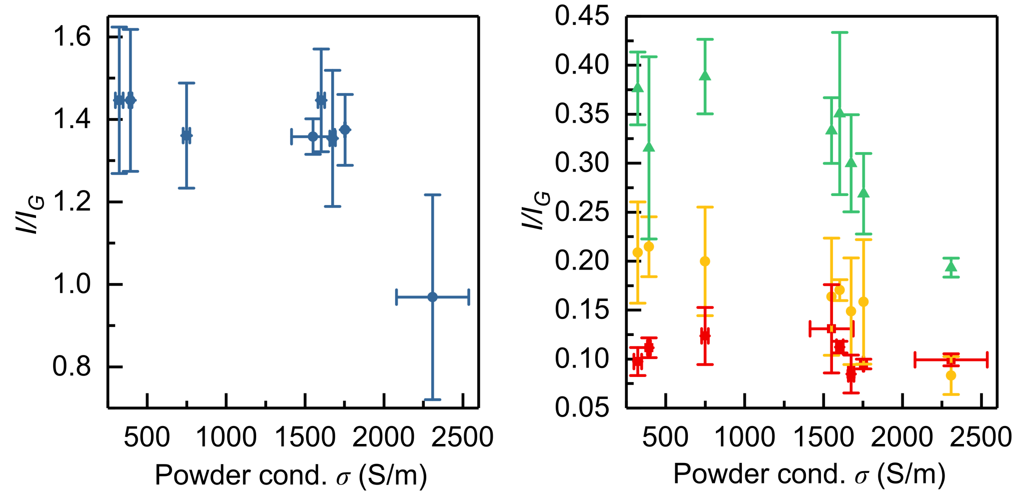

Figure S6. Fitted Raman results (average of three measurements) of thermal reduction samples reduced at increasing reduction temperature (compare Figure 4): left, the ratio of the D band to the G band against the measured powder conductivity σ is depicted; right, the ratio of the D* band (red), the D″ band (yellow) and the D′ band (green) to the G band against the measured powder conductivity σ is shown.

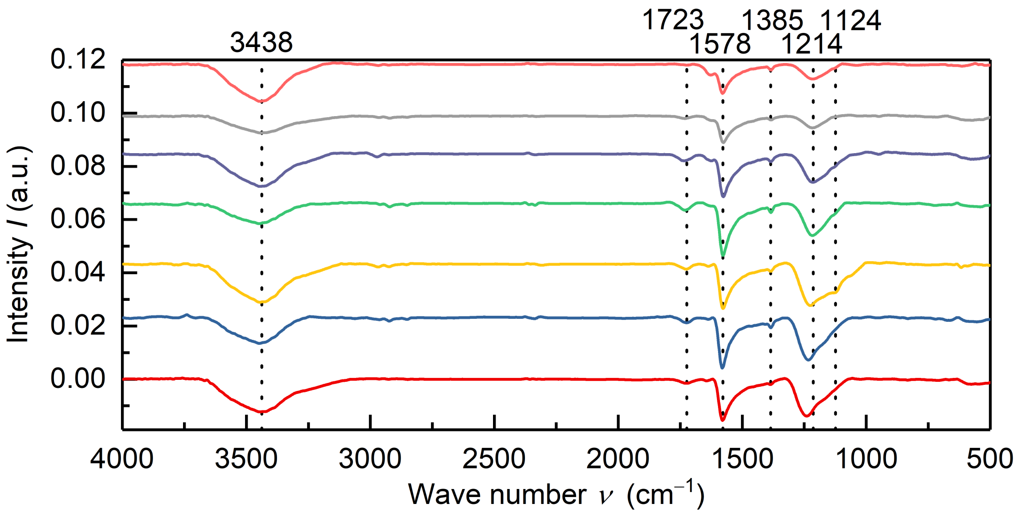

Figure S7. IR spectra of thermally reduced graphene oxide powders after reduction at different temperatures T_R in inert atmosphere for 30 min: graphene oxide educt (red) and powders reduced at 110 °C (blue, Run 26), 150 °C (yellow, Run 23), 250 °C (green, Run 24), 350 °C (violet, Run 13), 600 °C (grey, Run 7), 800 °C (salmon, Run 21).
