# Supplementary figures and images for: Development and Up-Scaling of Electrochemical Production and Mild Thermal Reduction of Graphene Oxide
Source: Materials (Basel). 2022 Jul 1;15(13):4639. doi: 10.3390/ma15134639 (PMC9267235; doi:10.3390/ma15134639)

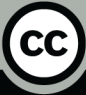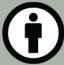

BY

Supplement: Supplementary file 1 [file materials-15-04639-s001.zip › Definitions/logo-ccby-eps-converted-to.pdf]

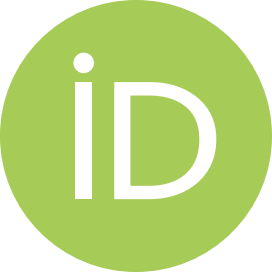

Supplement: Supplementary file 1 [file materials-15-04639-s001.zip › Definitions/logo-orcid-eps-converted-to.pdf]

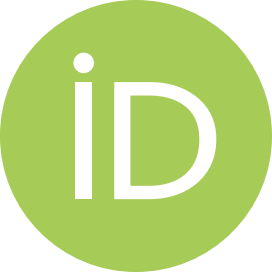

Supplement: Supplementary file 1 [file materials-15-04639-s001.zip › Definitions/logo-orcid.pdf]

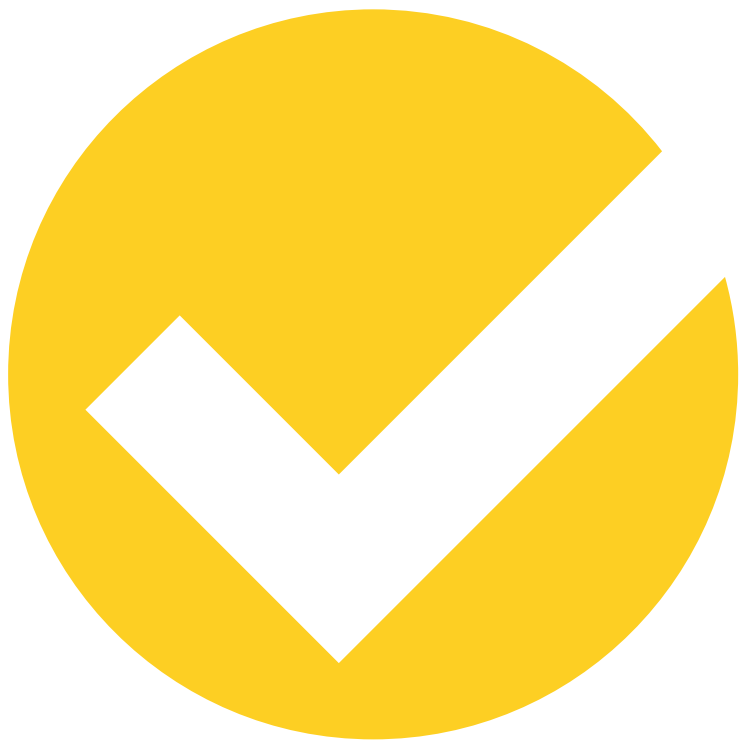

check for  
updates

Supplement: Supplementary file 1 [file materials-15-04639-s001.zip › Definitions/logo-updates.pdf]

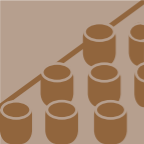

*materials*

Supplement: Supplementary file 1 [file materials-15-04639-s001.zip › Definitions/materials-logo-eps-converted-to.pdf]

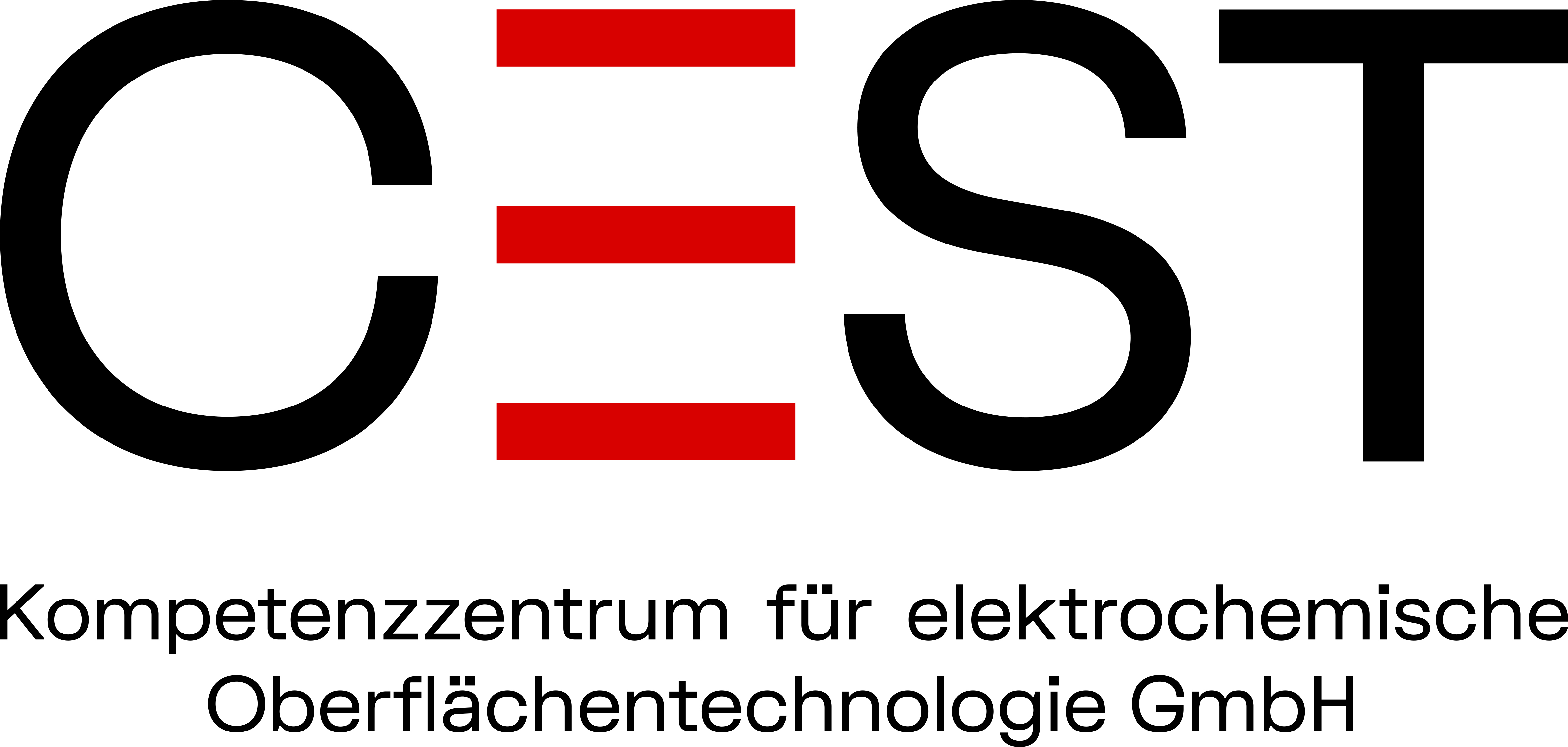

Supplement: Supplementary file 1 [file materials-15-04639-s001.zip › Figure/CEST_Logo_2022.jpg]

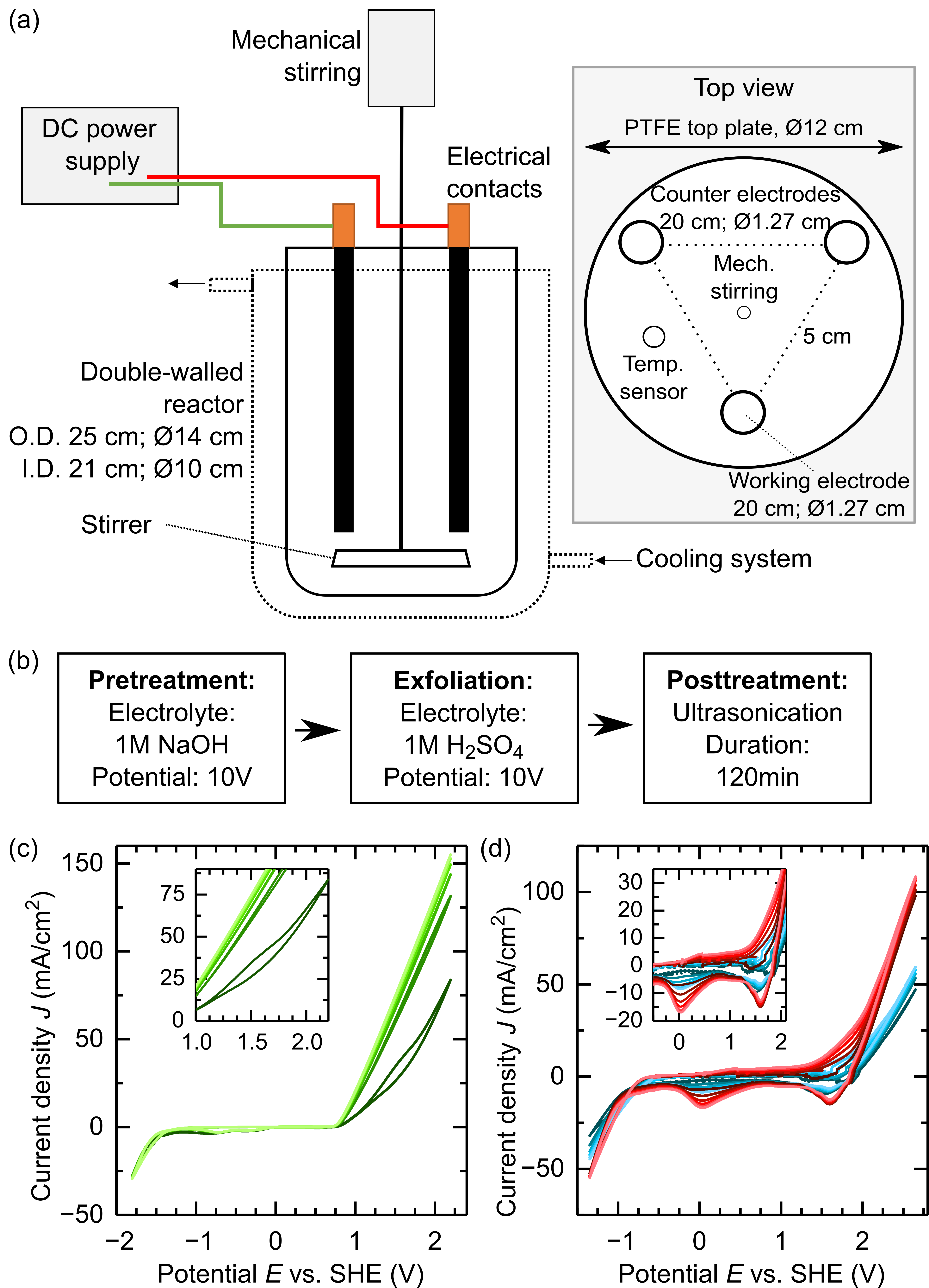

Supplement: Supplementary file 1 [file materials-15-04639-s001.zip › Figure/Figure1_insets_V2.png]

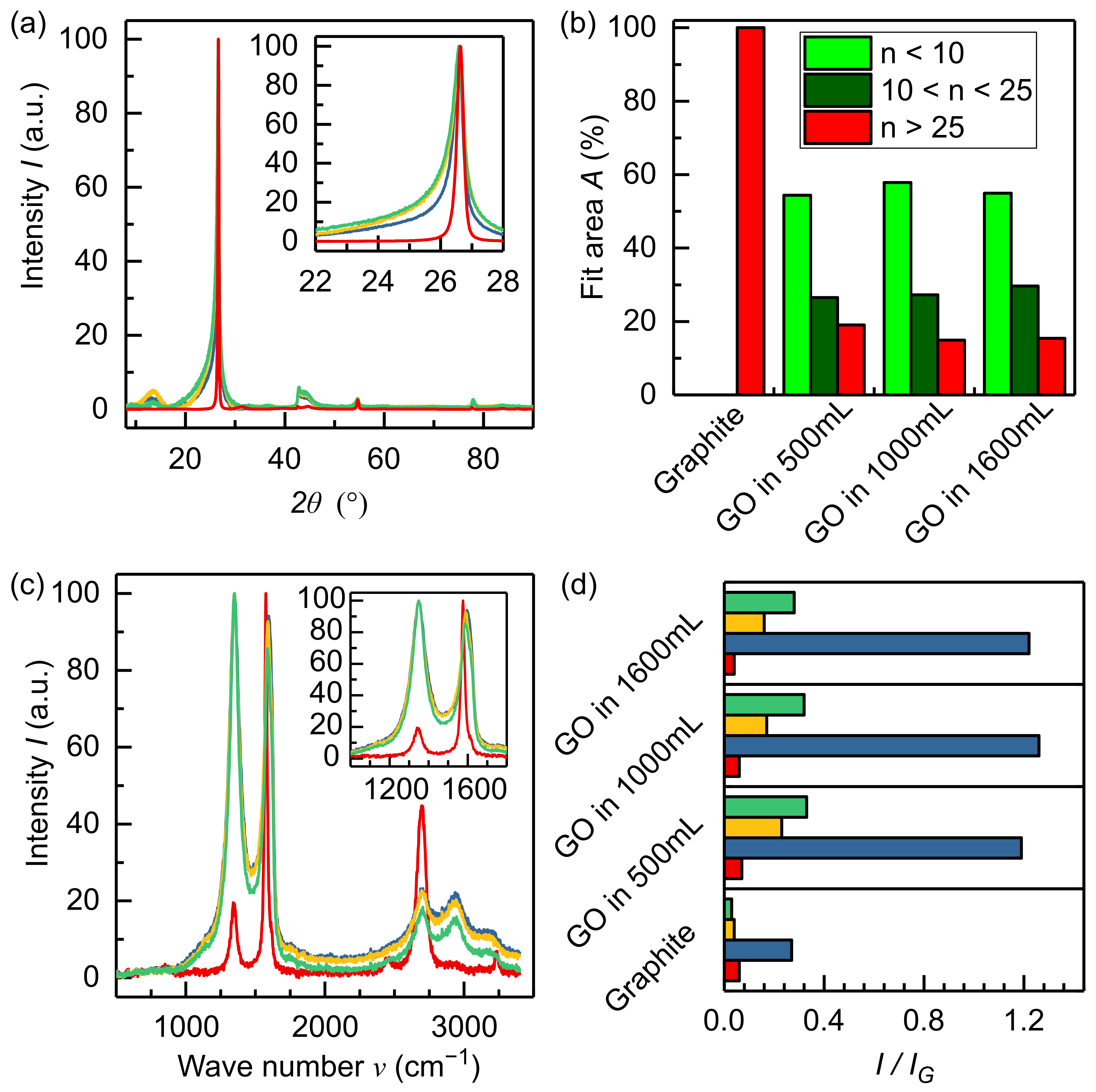

Supplement: Supplementary file 1 [file materials-15-04639-s001.zip › Figure/Figure2.png]

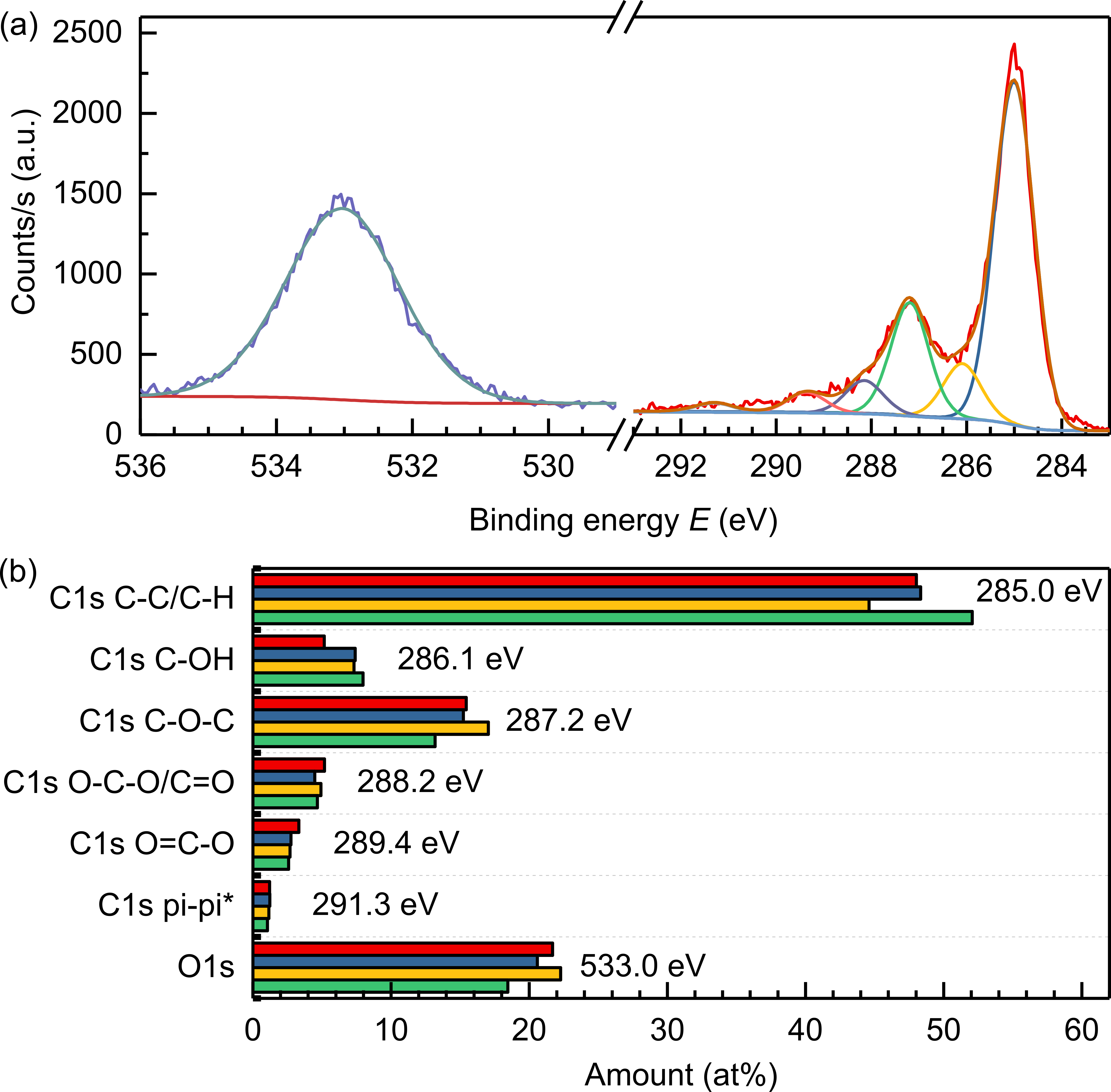

Supplement: Supplementary file 1 [file materials-15-04639-s001.zip › Figure/Figure3.png]

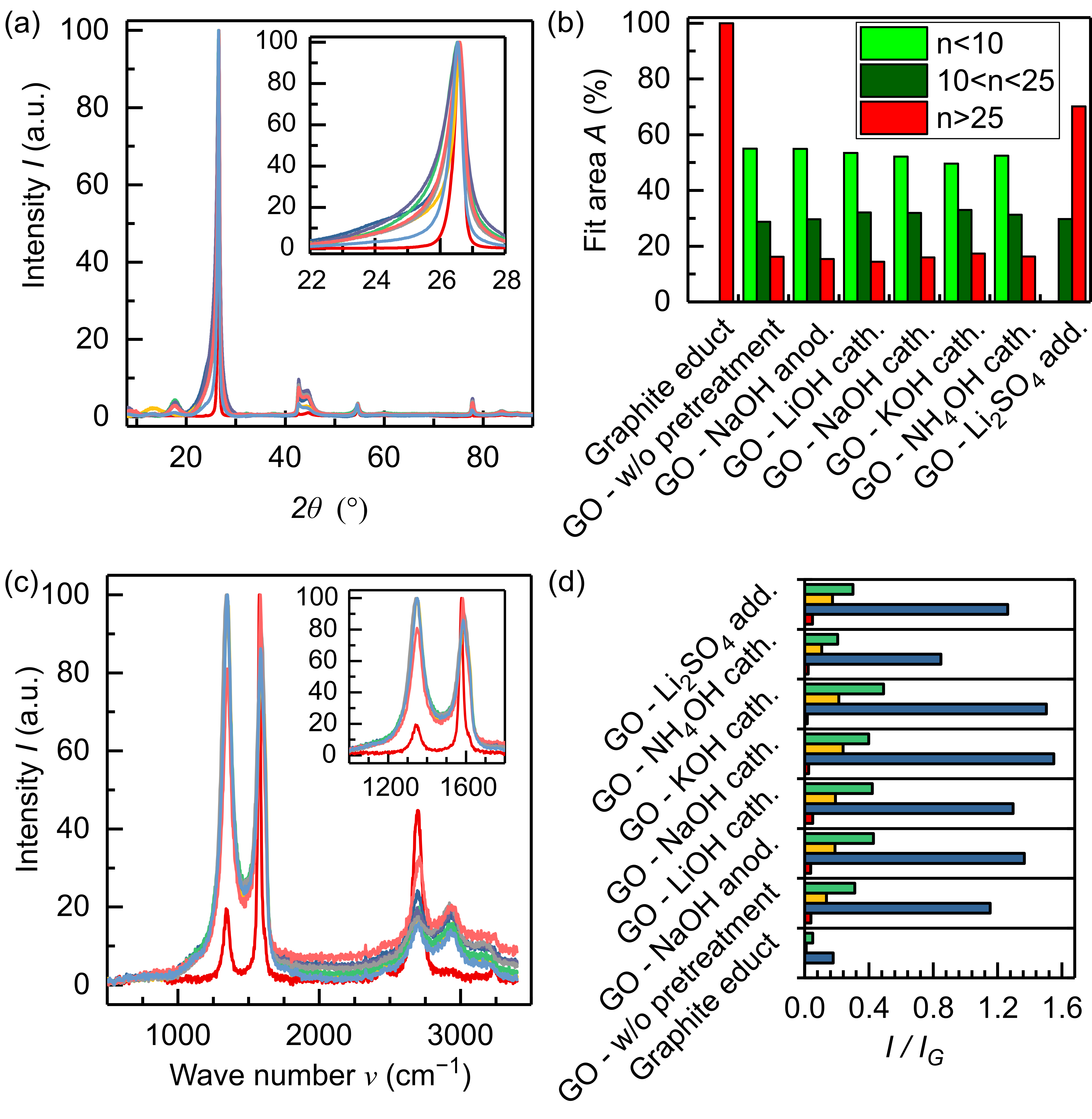

Supplement: Supplementary file 1 [file materials-15-04639-s001.zip › Figure/Figure5.png]

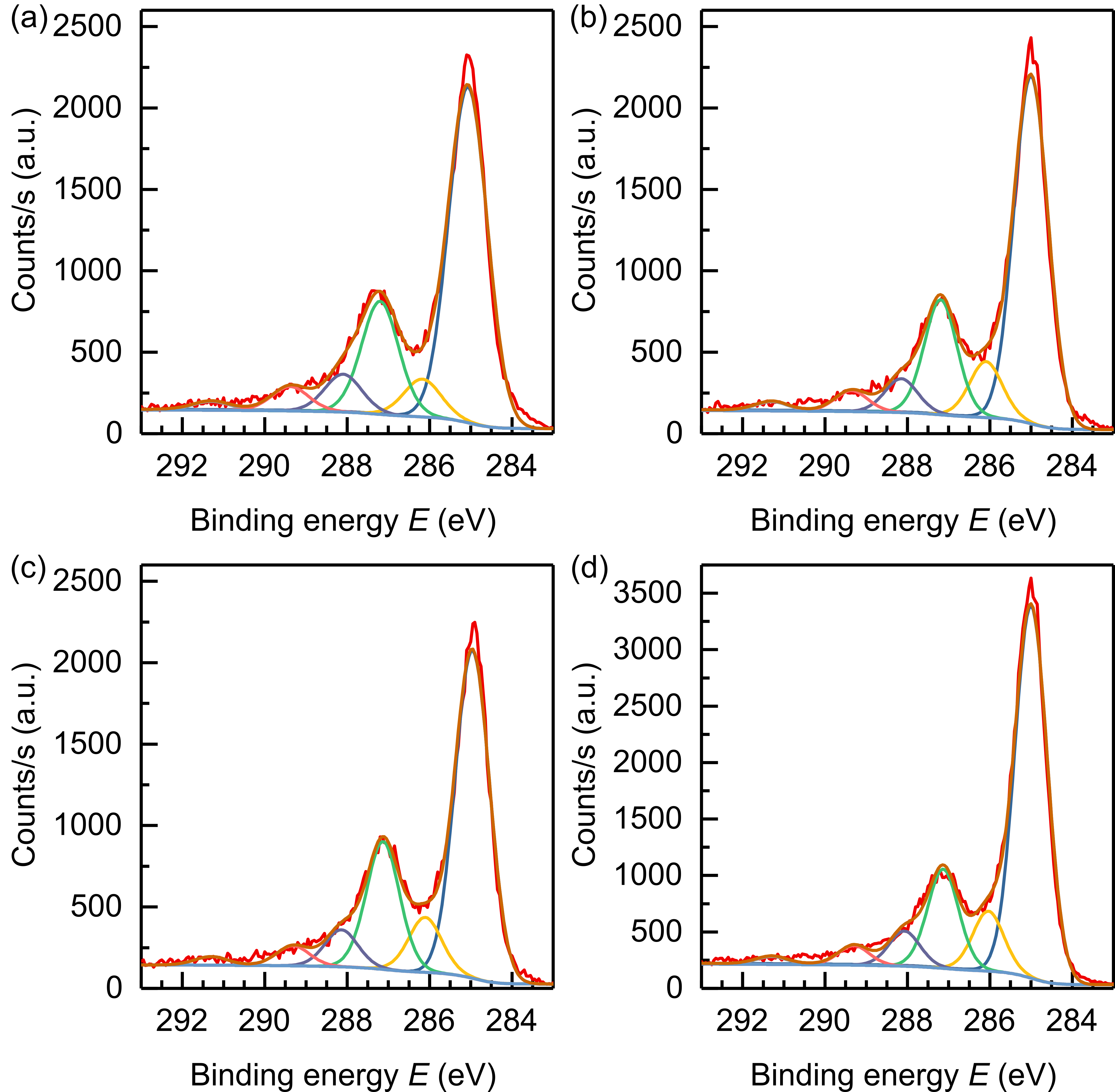

Supplement: Supplementary file 1 [file materials-15-04639-s001.zip › Figure/Figure6.png]

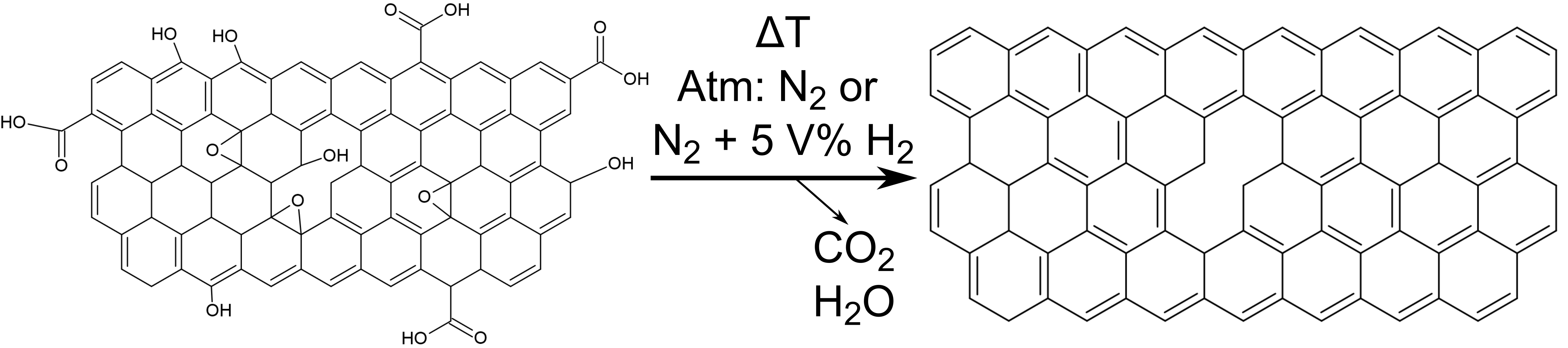

Supplement: Supplementary file 1 [file materials-15-04639-s001.zip › Figure/Figure_8.png]

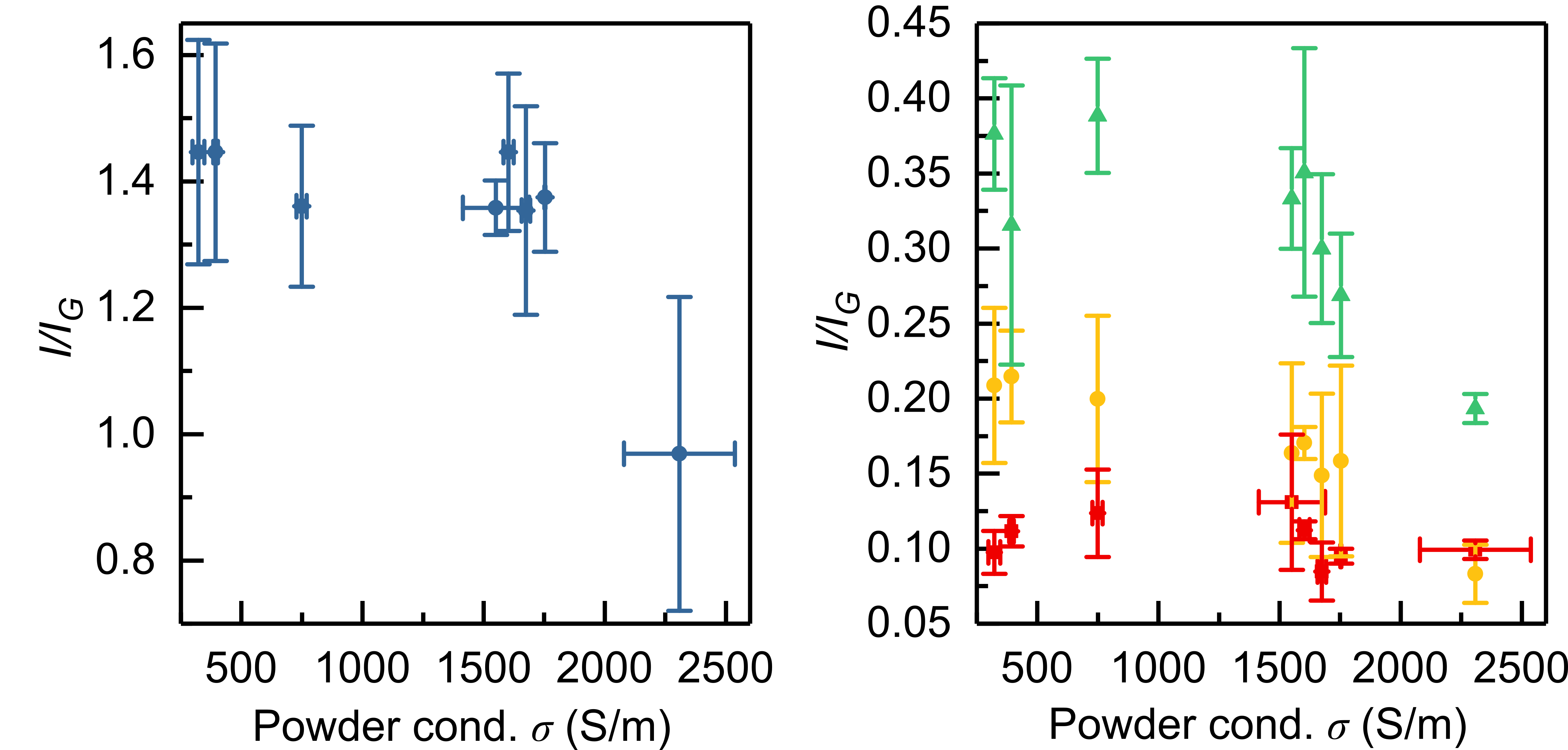

Supplement: Supplementary file 1 [file materials-15-04639-s001.zip › Figure/Figure_9_Raman_therm.png]

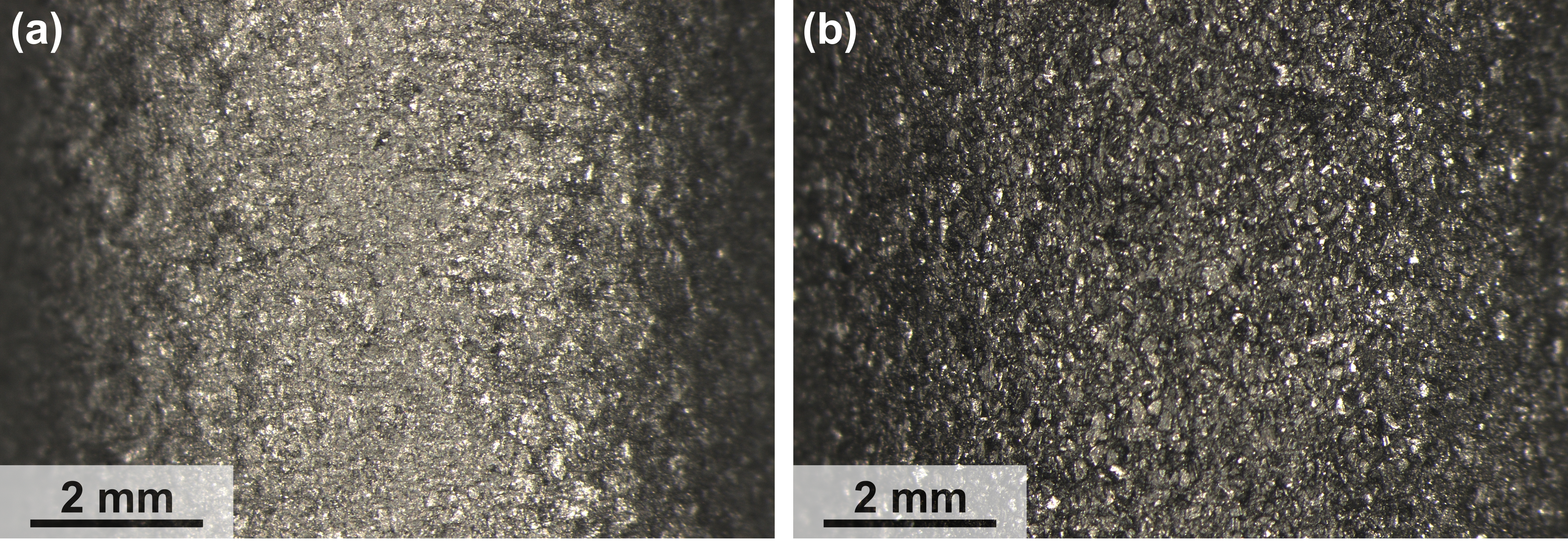

Supplement: Supplementary file 1 [file materials-15-04639-s001.zip › Figure/Figure_Graphite.png]

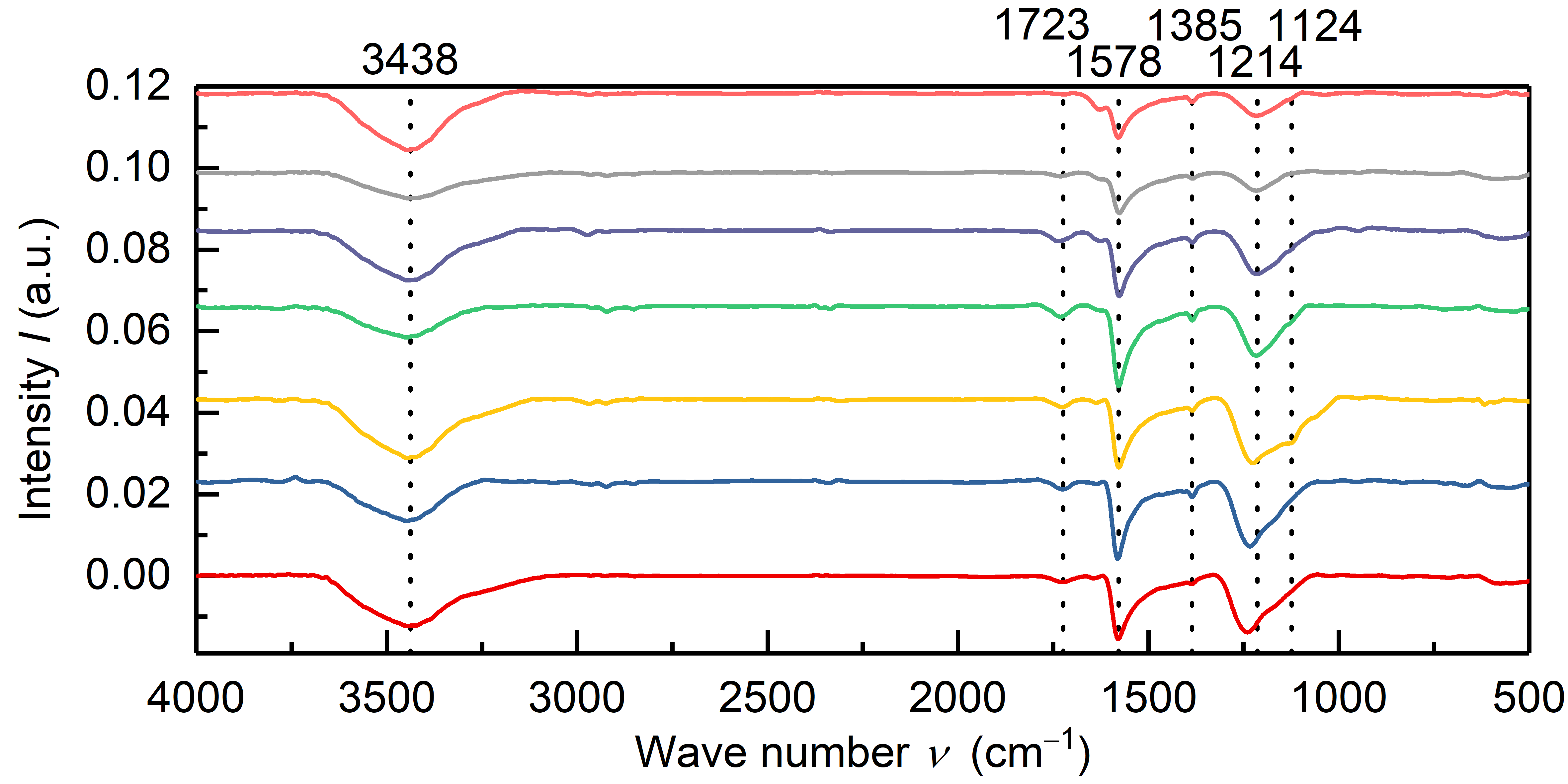

Supplement: Supplementary file 1 [file materials-15-04639-s001.zip › Figure/Fig_10_IR.png]

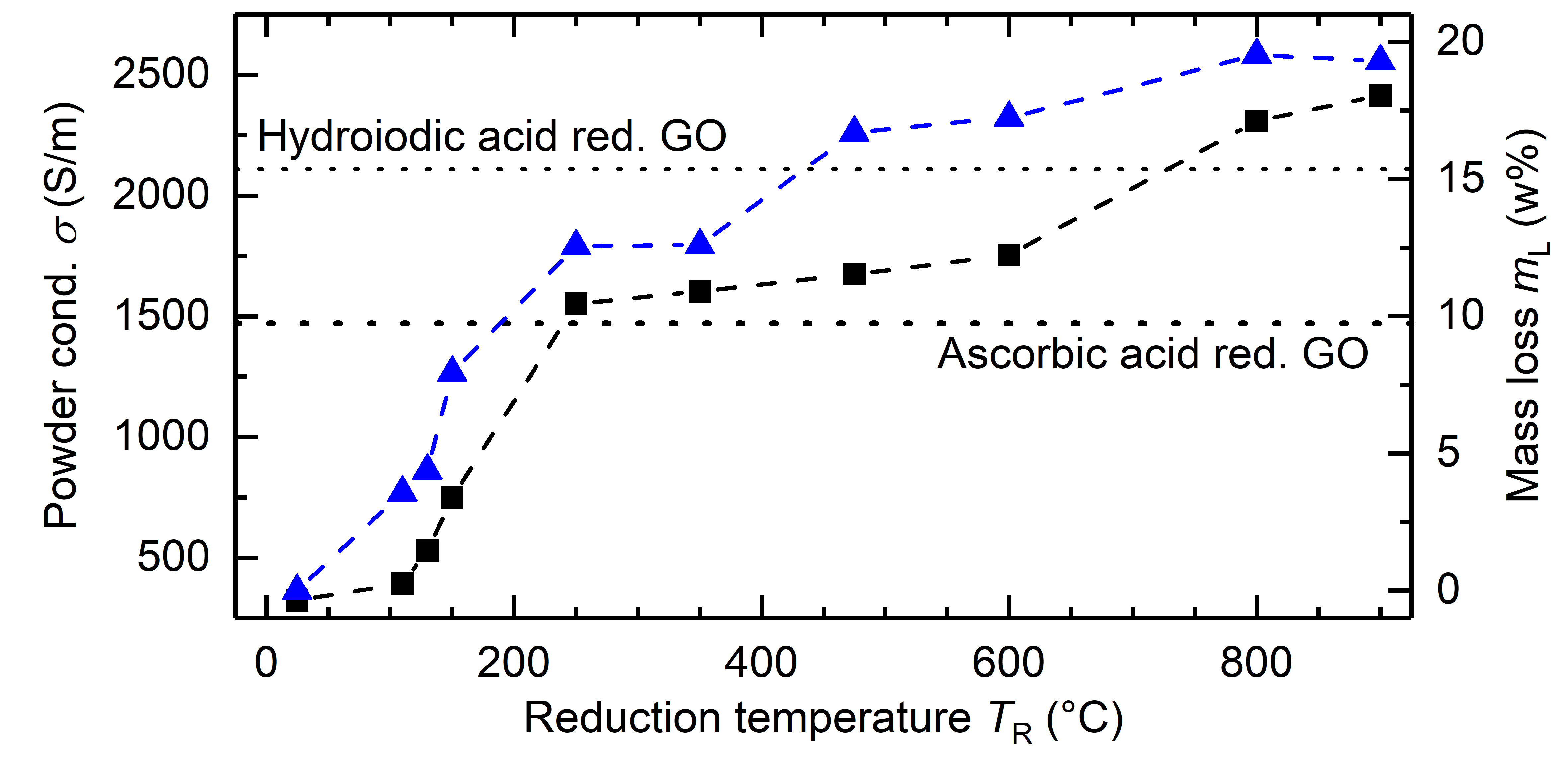

Supplement: Supplementary file 1 [file materials-15-04639-s001.zip › Figure/Fig_4.png]

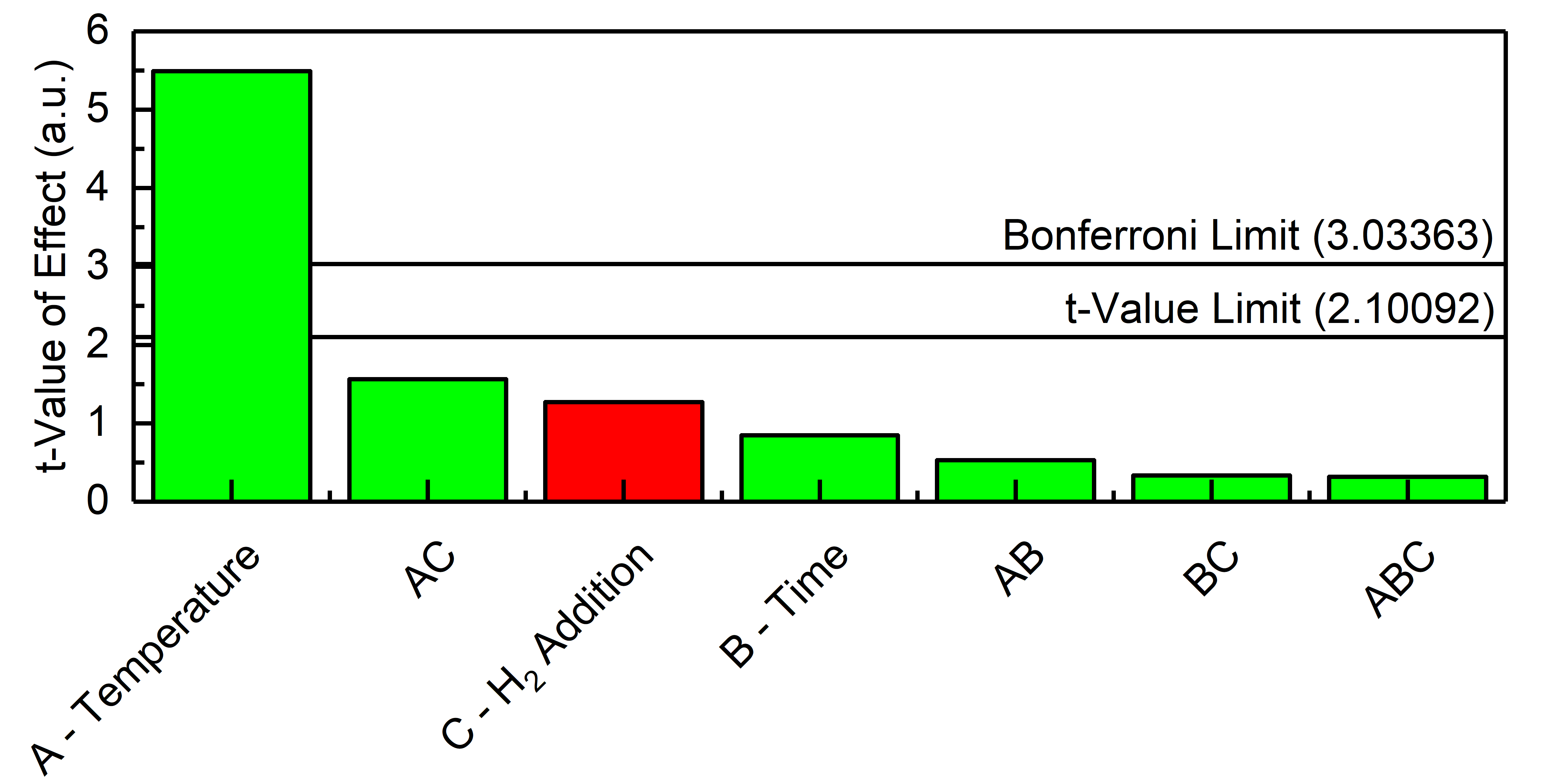

Supplement: Supplementary file 1 [file materials-15-04639-s001.zip › Figure/Fig_7_Pareto.png]

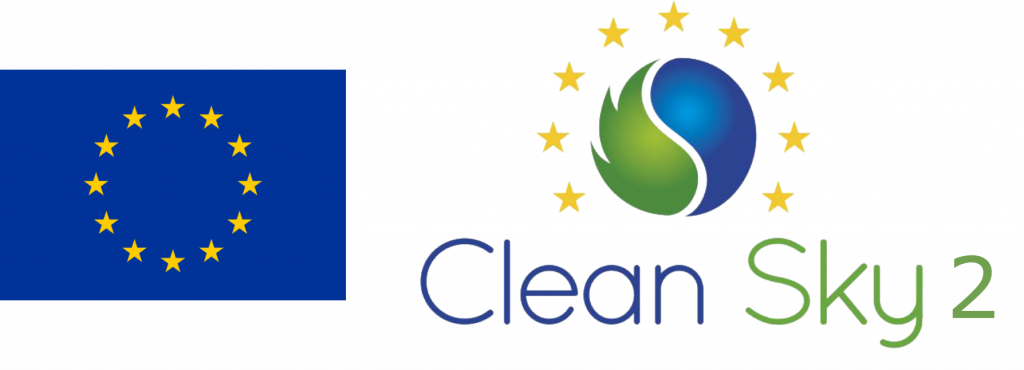

Supplement: Supplementary file 1 [file materials-15-04639-s001.zip › Figure/LOGOJU-1024x370.png]
